# Critical Gaps in Assisted Outpatient Treatment Research in the United States

**DOI:** 10.1007/s10488-024-01377-z

**Published:** 2024-04-30

**Authors:** Elizabeth Sinclair Hancq, Mark Munetz, Shanti C. Silver, Hope A. Parker, Natalie Bonfine

**Affiliations:** 1 Treatment Advocacy Center, Arlington, VA USA; 2 Northeast Ohio Medical University, Rootstown, OH USA

**Keywords:** Assisted Outpatient Treatment, Serious Mental Illness

## Abstract

In 2023, the White House included the implementation and improvement of assisted outpatient treatment in a list of under-researched strategies to support recovery and long-term treatment engagement for people with serious mental illness. Assisted outpatient treatment is a community-based, court-ordered, mental health treatment program for a subset of individuals with serious mental illness who have a history of difficulty adhering to treatment and staying well while living in the community. There is research supporting the use of assisted outpatient treatment for this specific population, however, the majority focuses on limited geographic regions, specific program organizations, and is outdated. Meanwhile, assisted outpatient treatment programs have increasingly been adopted by counties and states across the country. More research is needed to ensure that assisted outpatient treatment programs are being implemented in the most effective and equitable way possible. In this paper, the authors identify several key gaps in the current literature base relating to the effectiveness and implementation of assisted outpatient treatment.

## Introduction

The White House included assisted outpatient treatment (AOT) in the “Report of Mental Health Research Priorities,” released in February 2023, as one of the critical and timely opportunities in mental health research as part of a larger goal to foster long-term engagement in care and recovery for people with serious mental illness (Prabhakar & Rice, 2023). AOT is a community-based, court-ordered, mental health treatment program utilized in the United States for a subset of individuals with serious mental illness who have a history of involuntary hospitalizations, arrests, or demonstrated difficulty adhering to treatment and staying well while living in the community. There is evidence supporting the use of AOT for this specific population, however, there are significant limitations to this literature base.

First, the majority of existing AOT research focuses on a limited geographic region and is difficult to generalize to the entire United States. While there is a large body of research suggesting the effectiveness of outpatient commitment such as community treatment orders (Segal, 2024), AOT differs from most community treatment orders because it relies on the civil court system. This makes it difficult to directly compare its effectiveness with international models that do not involve a formal court order. Second, much of these data are now decades old and changing procedures and perspectives, such as the evolving role of AOT as a step down from inpatient hospitalization to a step up from outpatient treatment or as an alternative to competency restoration, have made it more difficult to compare current data to past research. Third, existing research fails to address many important questions about participant, program, and system-level outcomes. Despite this, AOT programs have increasingly been adopted by counties and states across the United States. Accordingly, more research is needed to inform best practices and ensure that programs are being implemented in the most effective, equitable, and least coercive way possible.

In this paper, we identify some of the most critical gaps in U.S-based AOT research relating to individual outcomes, program implementation, and impact on the healthcare system at large. When discussing outcomes, we also believe it is important to emphasize that these measures should not be limited to health service utilization, such as hospitalization episodes or medication adherence, but should also include recovery-oriented outcomes and the perspectives of participants themselves.

## Critical Research Gaps

### Individual Outcomes

One of the most important factors to consider in a mental health intervention is its ability to improve outcomes for participants. There is a wealth of evidence supporting the effectiveness of AOT programs across a variety of outcomes, including improving treatment adherence and the ability to care for oneself, while reducing hospitalization episodes, days hospitalized, arrests, victimization, and violence against self or others (Swanson et al., 2000; Swanson et al., 2001; Hiday et al., 2002; Esposito et al., 2008; Swartz et al., 2009; Gilbert et al., 2010; Phelan et al., 2010; Azar et al., 2018). However, this evidence comes from data that were collected more than a decade ago and are predominantly from New York State, making it difficult to determine if these results would be replicated in or applicable to the diverse variety of AOT programs implemented throughout the United States today.

There is also evidence that increased mental health treatment engagement may provide other health benefits for AOT participants, including earlier identification of physical health conditions (Segal et al., 2018). New research measuring individual-level outcomes should expand to include wellness and recovery-oriented outcomes, such as quality-of-life, perceived coercion, and feelings of empowerment, along with other outcomes identified and defined through community-based participatory research methods that include AOT participants in the design and development of research projects. New research should additionally determine if improvements are sustained following the completion of the AOT program.

However, it is not enough to demonstrate that AOT is effective at improving outcomes for participants. Court-ordered treatments like AOT should only be used for the subset of people with serious mental illness who may need them when voluntary treatment has failed. While there is evidence to suggest AOT participants have better outcomes than those in comparable voluntary services, this evidence is outdated (Swartz et al., 1999; Swartz et al., 2009; Busch et al., 2010; Gilbert et al., 2010).

There are also little data comparing the effectiveness of AOT among diverse populations. Data from New York showed that Black and Hispanic participants had worse outcomes following completion of AOT than white participants, including being more likely to be readmitted to a hospital (Van Dorn et al., 2010). Research into racial disparities in AOT is especially important given the overrepresentation of marginalized groups in involuntary treatment and AOT-eligible populations. Further, research into the effectiveness of AOT for special populations, such as those with criminal legal involvement, may also be relevant given recent proposals to utilize AOT as a diversion strategy*.* Additional populations of interest may include people with co-morbid substance use disorders, people experiencing chronic homelessness, and people who have been referred to AOT from different sources (e.g., clinicians, family members, etc.).

### AOT Program Implementation

While outcomes research conducted within an AOT program can indicate whether the program is working, research comparing outcomes across different types of programs is also needed to determine best practices for program implementation.

Judicial involvement is one element of AOT that varies substantially across programs. Some programs have limited judicial involvement whereas others use an “active court” model, which involves regular “check-ins” between participants, their treatment team, and the judge or magistrate. While the positive impact of compassionate judicial involvement,sometimes referred to as the “black robe effect,” is thought to be a part of what makes AOT programs effective (Munetz et al., 2014), there is no existing research that empirically examines this phenomenon. It is important to carefully examine how the level of judicial involvement impacts the efficacy of AOT programs and for which populations.

It is presumed that AOT is effective not only because it keeps an individual accountable to their treatment team, but also because it keeps the treatment system accountable to the individuals to whom they provide services. Therefore, it may be the case that AOT works because treatment teams are required to ensure that program participants do not “fall through the cracks” while in the program. However, systems of accountability, such as employing an AOT monitor or requiring meetings between the treatment team and the court, can vary from program to program, and no research has yet investigated which mechanisms are most effective. Future research is needed in this area to understand best practices for program implementation and which different system accountability mechanisms are the most successful at improving participant and program outcomes.

### Impact on Healthcare System

AOT has also been thought to decrease the public cost of serious mental illness through decreasing the need for inpatient hospitalization and/or diverting individuals away from the criminal legal system into treatment. Prior research has shown that among individuals with a history of criminal legal system involvement, AOT can reduce arrests by nearly two-thirds in any given month and reduce the chance of being arrested for a violent offense by 88% (Gilbert et al., 2010; Link et al., 2011). In addition, AOT has been shown to reduce hospitalizations by up to 85% and reduce days spent in the hospital by 44% (Esposito et al., 2008; Swartz et al., 2009; Azar et al., 2018). The positive impacts of these reductions may extend to entire systems of care, such as through improved, targeted allocation of available public hospital beds to those most in need. However, as with other gaps in our knowledge of AOT, most of this research comes out of New York State and further research is needed to see if this effect can be generalized to AOT programs nationwide. Additionally, there is limited research examining how community and mental health system contextual factors such as the availability of intensive outpatient services or behavioral workforce shortages may impact the effectiveness or implementation of AOT programs. Multi-site or multi-state program comparison research examining the community-impact of AOT programs is needed to further understand the community and system impacts of AOT programs.

## Conclusion

Many of the gaps in AOT literature are not unique and exist for many widely implemented mental health interventions. However, expanding our knowledge of the impact of AOT in the United States at the individual, program, and systems levels is paramount because AOT is a court-ordered treatment program and should only be utilized in the specific populations for whom it is necessary and beneficial.

To further include diverse opinions and perspectives, we highlight the need for AOT research to include the perspectives and voices of AOT participants and their families as their insights are instrumental in helping to shape recovery-oriented and person-centered programs. We call on research institutions, funders, and publishers to respond to the call of the White House’s “Report on Mental Health Research Priorities” and prioritize the further investigation of the practice of AOT for people with serious mental illness.
